# 

**Published:** 1891-09-05

**Authors:** 


PRICE ONE PENNY.

No. 258, Vol. X.—September 5th, 1891.

[Registered at the General Post Office as a Newspaper for transmission in the United Kingdom and Abroad.]

WITH EXTRA NURSING SUPPLEMENT.

Per Annum, 6s. 6d.; post free.

Monthly Parts, 6d.; post free 8d.

Ditto per Annum, 8s., post free.

### THE HOSPITAL

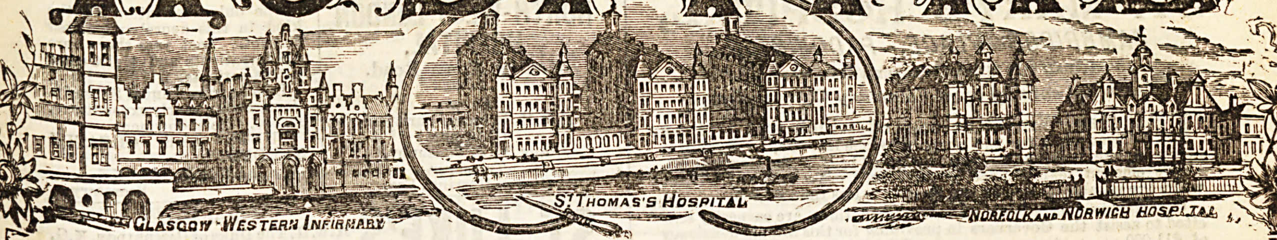

A WEEKLY JOURNAL OF
Science, Medicine, Nursing and Philanthropy.

### SATURDAY, SEPTEMBER 5th, 1891.

**The Position of the Provincial Medical Schools** - 265

**Passing Topics.**—Shall Nurses Cease to be Lay Women?—I. 266

**The Doctor's Armchair.**—The Bodies and Minds of Children - 267

**The Story of the Insane from Year to Year** - 268

**Everybody's Page** - 269

**Notes and News** - 270

**General Charities—Scraps and Gleanings** - 271

**Annotations.**—Sane Expenditure—The Vaccination Controversy—Alas! Poor Genius - 272

**The Student of Medicine.**—Syphilis: Its Transmission and Treatment—Appointments—Vacancies—Scholarships and Prizes

**The Practitioner's Mirror and Retrospect—**

MEDICINE.—Some Points in the Treatment of Diabetes—I. 273

SURGERY.—Diseases of the Fingers (*continued*). E—Diseases of the Tendons, 273; Nævoid Lipoma and its Association with Hypertrophy of the Toes - 274

OPHTHALMOLOGY.—Ametropia and the Cardio-Vascular System - 275

NEW DRUGS, APPLIANCES, AND THINGS MEDICAL.—At the British Medical Association. Section A (*continued*), 275; Antiseptic India-Rubber Cushions for Bed Pan, Bed Slipper and Bed Baths - 276

**Editor's Letter-Box.**—Dr. A. R. Wallace and the Vaccination Commission - 276

### EXTRA SUPPLEMENT—THE NURSING MIRROR.

**En Passant.**—The Liverpool Nurses—The Birmingham Nurses' Co-operation—The Coventry and Warwick Hospital—For the Adults—The Nurse and the Medicine Bottle—Nursing Association for Waplington - cxxxi

**Lectures on Surgical Ward Work and Nursing.** By ALEX. MILES, M.D. Edin., F.R.C.S.E. XXXIV.—Surgical Instruments - cxxxii

**A Few Words to Nurses** - cxxxii

**The Princess of Wales and the Nurses** - cxxxiii

**For Reading to the Sick**—To Convalescents - cxxxiii

**Bible Women** - cxxxiv

**Notes and Queries** - cxxxv

**Presentations** - cxxxv

**Everybody's Opinion.**—Outdoor Uniforms - cxxxv

**Appointments** - cxxxv

**Off Duty.**—A Little Leader - cxxxvi

**"A 'Harbour of Rest' in the Land of Old Age"** - cxxxvi

**Amusements and Relaxation** - cxxxvi

